# Analysis of errors in the structure determination of MsbA

**DOI:** 10.1107/S0907444909001292

**Published:** 2009-01-20

**Authors:** Philip D. Jeffrey

**Affiliations:** a Department of Molecular Biology, Princeton University, Princeton, NJ 08544, USA

**Keywords:** protein crystallography, methodology

## Abstract

The determination of incorrect structures for the ABC transporter MsbA gave rise to questions of how this could have occurred. Methodological aspects of the MsbA structure determination are explored in light of this error.

## Abstract 2

An analysis is presented of the methodological errors that led to the incorrect structure of MsbA.

## 1. Introduction

Evidence of significant errors in the crystal structures of the transporters MsbA and EmrE (Dawson & Locher, 2006 ▶; Tate, 2006 ▶) led to the retraction of five structure papers (Chang *et al.*, 2006 ▶; Chang, 2007 ▶; Ma & Chang, 2007 ▶), the so-called ‘great pentaretraction’ (Miller, 2007 ▶). These structures corresponded to Protein Data Bank (PDB; Berman *et al.*, 2000 ▶) entries 1jsq, 1pf4 and 1z2r for MsbA, and 1s7b and 2f2m for EmrE, which were moved to the PDB obsolete archive. These retractions generated much discussion (Miller, 2006 ▶, 2007 ▶; Petsko, 2007 ▶; Matthews, 2007 ▶; Jones & Kleywegt, 2007 ▶), at least in part because three of the retracted papers were published in a prominent journal in the field of structural biology (Chang & Roth, 2001 ▶; Chang, 2003 ▶; Reyes & Chang, 2005 ▶; Ma & Chang, 2004 ▶; Pornillos *et al.*, 2005 ▶).

In an attempt to understand how such incorrect structure determinations could occur, examples and conclusions will be presented based on test data and the published structure papers. MsbA and EmrE are unrelated in structure but they shared the same pathology during structure determination and the conclusions apply to both structures. The corrected structures were published in late 2007 (Chen *et al.*, 2007 ▶; Ward *et al.*, 2007 ▶).

## 2. The initial error

It has been indicated (Chang *et al.*, 2006 ▶) that the initial error in the structure determinations was the accidental inversion of the sign of the anomalous difference in a data-conversion step that converted experimental intensity (*I*) values to structure-factor moduli (*F*). Specifically, *I*(*h*, *k*, *l*) and *I*(−*h*, −*k*, −*l*) were converted to |*F*(−*h*, −*k*, −*l*)| and |*F*(*h*, *k*, *l*)|, respectively. This conversion utilized an in-house program for which the source code was unavailable (G. Chang, personal communication).

The anomalous difference (Δ_ano_) can be expressed as |*F*(*h*, *k*, *l*)| − |*F*(−*h*, −*k*, −*l*)|. Negation of this anomalous difference is equivalent to a centrosymmetric misassignment of the Miller index from (*h*, *k*, *l*) to (−*h*, −*k*, −*l*), changing the hand of the reciprocal-lattice indexing. This is unlikely, but possible, during the data-processing step by a misstatement of the detector or goniostat geometry.

In experimental SAD or MAD phasing the combination of inverted anomalous data with the correct heavy-atom sub­structure leads to an uninterpretable map, as does combination of the centrosymmetrically inverted substructure with anomalous data of the correct sign (Matthews, 2007 ▶). How­ever, it is possible to obtain a superficially interpretable map, albeit a centrosymmetrically inverted one, if an inverted heavy-atom substructure is matched with data with an inverted anomalous sign (Wang *et al.*, 2007 ▶).

## 3. MAD phasing examples

The consequences of inverting the sign of the anomalous signal during phase determination have been discussed by Matthews (2007 ▶) with reference to the MsbA and EmrE cases. To test how far one might practically proceed with data sets that had inverted anomalous signals, two example MAD data sets at high and low resolution were selected. In both cases the *SHELX* program suite (Sheldrick, 2008 ▶) was used to determine heavy-atom locations and calculate experimental phases. A simple program was written to invert the anomalous sign in otherwise unmodified files output by *SCALEPACK* (Otwinowski & Minor, 1997 ▶) prior to using *SHELXC*.

In this protocol *SHELXC* was used to calculate an improved magnitude estimate of the substructure structure factor *F*_A_, *SHELXD* determined the heavy-atom substructure and *SHELXE* calculated phases from the substructure and the MAD data to produce phases corresponding to an electron-density map modified by solvent flattening. The heavy-atom substructure determined by *SHELXD* is inherently ambiguous, with two possible solutions being equally consistent with the data: one with coordinates at (*x*, *y*, *z*) and the other with centrosymmetrically inverted coordinates at (−*x*, −*y*, −*z*). In favorable cases there is a clear distinction between the correct solution and the incorrect solution during the phasing process in *SHELXE*, with one heavy-atom substructure producing an interpretable map and the other producing a nonsense map.

### 3.1. MAD phasing at high resolution

The DED domain of MC159 was a straightforward 1.8 Å resolution multiwavelength anomalous dispersion (MAD) structure determination using selenomethionine-labeled protein (Li *et al.*, 2006 ▶). MC159 crystals grew in space group *P*2_1_2_1_2_1_, with unit-cell parameters *a* = 35.10, *b* = 63.50, *c* = 76.46 Å. MAD phasing yielded a very interpretable experimental map that was amenable to automatic building using *ARP*/*wARP* (Perrakis *et al.*, 1999 ▶). *SHELX* was used to prepare the data, find the heavy-atom substructure of four seleniums, generate experimental phases and perform solvent flattening with an assumed solvent content of 35% by volume. The same phasing protocol was run using the original data and using the same data with inverted anomalous signal. The results are summarized in Table 1 ▶.

**Figure 1:**
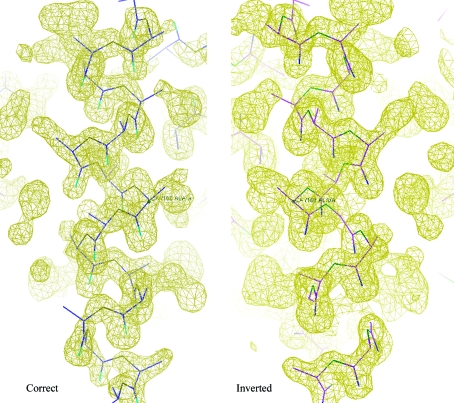
Comparison of the MC159 experimental maps derived from data with and without inversion of the anomalous signal. Atomic models fitted to the inverted map correspond to d-amino acids.

The anomalous signal statistics in *SHELXC* were essentially identical for the unmodified and inverted data because the magnitudes of the anomalous differences were maintained; only their sign was altered. During the heavy-atom substructure-determination step in *SHELXD* the correlation coefficient for weak reflections was similar in both cases, as were both the contrast and pseudo-free correlation coefficients during the phasing and solvent-flattening steps in *SHELXE*. These statistics are often those that prove to be most useful for monitoring success in experimental phasing. For these statistical values, the correct and inverted anomalous sign data were almost indistinguishable. Table 1 ▶ shows the results for *SHELXE* runs using the sites found by *SHELXD* with and without inversion applied, as there is an inherent centrosymmetric ambiguity in the sites located by *SHELXD*. The heavy-atom substructures corresponding to unmodified and inverted data were found to be related by inversion after compensation for an alternative origin choice and a crystallo­graphic symmetry operator. They were also superimposible and therefore the heavy-atom substructures were consistent apart from the inversion. The experimental phases led to electron-density maps (Fig. 1 ▶) that were essentially identical in terms of quality except that they were centrosymmetrically inverted with respect to each other.

This situation was not found to be unique to the use of *SHELX* for experimental phasing and this same behavior could be reproduced with *SOLVE*/*RESOLVE* (Terwilliger & Berendzen, 1999 ▶; data not shown).

### 3.2. MAD phasing at low resolution

The structure determination of the intramembrane protease S2P (Feng *et al.*, 2007 ▶) was an example that was somewhat more representative of the lower resolution of the MsbA and EmrE studies. S2P crystals formed in space group *R*3 and were indexed in the hexagonal setting *H*3 with unit-cell parameters *a* = *b* = 123.86, *c* = 136.47 Å. Experimental MAD data to a maximum resolution of 3.8 Å were collected from selenomethionine-labeled protein. The same protocol was used to generate experimental phases for S2P as was used in the MC159 example above. While the 3.8 Å resolution MAD map predictably lacked the clarity of the 1.8 Å resolution map of MC159, the hand of the helices and many of the side chains are clearly visible in the map. If the same phases are used to calculate a map at the 4.5 Å resolution of the original *Escherichia coli* MsbA study (Chang & Roth, 2001 ▶) the hand of the helices becomes very difficult to detect.

The results of MAD phasing are summarized in Table 1 ▶ and as with the higher resolution example there was no distinction on statistical criteria alone between solutions based on data with the correct and inverted anomalous signs. Use of the program *SHARP* (de La Fortelle & Bricogne, 1997 ▶) considerably improved the interpretability of the electron-density maps from both the correct and inverted anomalous data with similar phasing statistics (data not shown). The experimentally phased electron-density maps were similar in superficial interpretability, including the detection of α-helices. Since the heavy-atom substructure from the inverted anomalous signal was the centrosymmetric inverse of the corrected anomalous data, the phases from one solution were the approximate negative of the other. The experimental maps were also the centrosymmetric inverse of each other.

### 3.3. Conclusions from the test cases

The use of conventional phasing protocols and programs on data with an inverted anomalous sign gives rise to phases that have essentially the same statistical quality indicators as phases derived from data with the correct sign. This is unlikely to be influenced by choice of program employed or changes in phasing protocol beyond the initial inversion event. The experimental phasing process applied to the inverted anomalous data first gives rise to a heavy-atom substructure determination that is centrosymmetrically inverted with respect to the correct one. Combining the inverted data with the inverted substructure during the phase-calculation step leads to phases that are the negative of the correct ones (‘inverted phases’) in the absence of obscuring factors such as alternative choices of origin.

It is noteworthy that the inverted anomalous data and corresponding inverted phases are self-consistent: phased anomalous difference maps gave rise to positive peaks at the inverted substructure locations. Along parallel lines, it also proved possible to solve a structure by molecular replacement in the contrived case of having a centrosymmetrically inverted structure of S2P as a model with essentially the same log-likelihood gain and *Z*-score statistics using *Phaser* (Storoni *et al.*, 2004 ▶; data not shown).

## 4. Compounding the error

Although one can obtain a superficially interpretable electron-density map from phases derived from data with an inverted anomalous signal, this map will resist interpretation in terms of protein structure of conventional geometry. It would be difficult not to detect this error in high-resolution cases with high-quality experimental phases. In the retractions (Chang *et al.*, 2006 ▶; Chang, 2007 ▶; Ma & Chang, 2007 ▶) the authors identified two errors with the structures: the model was fitted into an inverted map with a model of non-inverted geometry and connectivity errors in the interpretation led to incorrect topology.

### 4.1. Chirality

The relatively low resolutions of the native data used in MsbA refinement (4.5 and 4.2 Å for *E. coli* and *Salmonella typhimurium*, respectively) made detection of the map inversion error difficult. Chang & Roth (2001 ▶) characterized the experimental MsbA phases as having ‘…yielded electron-density maps of excellent quality for tracing a polypeptide chain’ and the reported figure of merit for the phases was 0.7. Noncrystallographic symmetry averaging and sharpening of the data were apparently insufficient to resolve features that would have indicated that the map was calculated on the incorrect hand.

**Figure 2:**
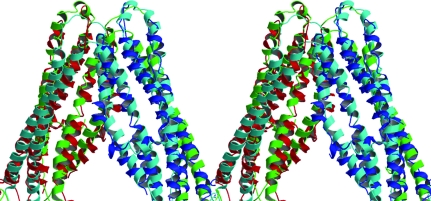
Stereo image of the superimposition of correct and incorrect *E. coli* MsbA structures. A part of the asymmetric unit where two monomers interact is shown. Two polypeptide chains of the correct model are colored cyan and green. The incorrect model has been inverted to put the model in the same hand as the correct structure, with chains colored red and blue. This figure was constructed using *MOLSCRIPT* (Kraulis, 1991 ▶) and *RASTER*3*D* (Merritt & Murphy, 1994 ▶).

Models with conventional geometry were built into electron-density maps that were inconsistent with this geo­metry. The incorrect structure can be compared with the correct one upon centrosymmetric inversion of the incorrect structure to put them both in the same hand. Comparison of these structures show that the locations of many of the secondary-structure elements were comparable, allowing inversion. Although the locations of the helices correspond, the polypeptide backbones do not overlay (Fig. 2 ▶).

### 4.2. Sequence assignment

Each of the structures of MsbA and EmrE had experimental MAD or SAD data associated with them; however, these were obtained by soaking osmium, mercury or arsenic compounds into the crystals. There were no data from selenomethionine-labeled protein. Selenomethionine can greatly assist sequence assignment and can be a sensitive test for topology errors (Hunte *et al.*, 2005 ▶). Broken density in the loops that interconnected the secondary-structure elements also gave rise to incorrect topology connections (Chang *et al.*, 2006 ▶). As an incorrect model was being built into an inverted map, it is not surprising that the topology would also be built incorrectly, making a correct sequence assignment impossible.

### 4.3. Refinement

The models of MsbA and EmrE were built into inverted maps and with incorrect topology and therefore essentially all the atom locations were wrong. Optimization of the model during refinement would at best fit the low-resolution features of the map with cylindrical helix density from the model overlapping that of the inverted map. Higher resolution details arising from detailed atomic positions would be impossible to reproduce accurately.

This was reflected in the relatively high free *R* factors for the refined structures as conventional single-model representations for MsbA and EmrE (Table 2 ▶). This behavior was rationalized as being a consequence of intrinsic crystal disorder (Chang & Roth, 2001 ▶) and multicopy refinement (Pellegrini *et al.*, 1997 ▶) was used in refinement to reduce the free *R* factor. Multicopy refinement replaces the conventional single-model description of a protein structure with an ensemble of non-interacting copies of the model in order to better fit the experimental data. This method has the potential to model disorder and motion not amenable to the single-model description of electron density. How­ever, for an *N*-copy multicopy refinement the observation-to-parameter ratio is decreased *N*-fold. For the structure of *E. coli* MsbA an ensemble of 16 copies of the asymmetric unit were refined simultaneously, with non­crystallographic symmetry restraints applied within each set (Chang & Roth, 2001 ▶). Since observation-to-parameter ratios are already particularly poor at 4.5 Å resolution, this made a bad situation considerably worse and led to overfitting. Chen & Chapman (2001 ▶) have indicated that multicopy refinements show signs of overfitting even at significantly higher resolutions.

Table 2 ▶ shows that when multicopy refinement was employed with *E. coli* MsbA the difference between the working-set *R* factor and test-set *R* factor increases from 7 to 11%, but the free *R* factor does decrease by 7%. Although the multicopy refinement represents a better model for the data as atoms are allowed to wander away from the single-model representation, it was not realised at the time that this was because the structure was wrong instead of modelling crystal disorder. The relatively large average deviation of the multicopy models from the mean atomic position is illustrated in supplementary Fig. 1 in Chang & Roth (2001 ▶).

Another factor that biased the free *R* factor was the selection of the test-set reflections in a random manner. In cases where there is significant noncrystallographic symmetry, the random choice of the free *R*-factor set introduces relationships between the working set and test set that depend on the number of molecules related by noncrystallographic symmetry and the extent to which they are similar to each other (Fabiola *et al.*, 2006 ▶). This cross-talk biases the free *R* factor to lower values and can be reduced by selecting test-set reflections in shells of constant resolution. However, it is not a simple matter to quantify the magnitude of this effect.

The third factor was the systematic omission of weak data during refinement by applying a 2σ(*F*) cutoff to the data. This is illustrated by the *R* factors reported for the corrected structures of MsbA (Ward *et al.*, 2007 ▶) refined with either 2σ(*F*) or 0σ(*F*) cutoffs. *R* factors were reduced by up to 4%, while at the same time up to 32% of the weakest reflections were discarded (Table 3 ▶). Although the structures had superficially better agreement with the data that remained, it is likely that they were correspondingly less accurate by failing to incorporate the measured weaker data in refinement.

## 5. Detecting wrong structures at low resolution

The free *R* factors of the single-model refinements of MsbA (Table 2 ▶) were high enough to warrant skepticism as to the accuracy of the structures. The free *R* factor is of great utility in assessing the overall quality of macromolecular structures (Brünger, 1997 ▶), but it is not foolproof. Use of multicopy refinement and a random choice of test-set reflections reduced the *R*_free_ to a value that was not unreasonable considering the resolution. The free *R* factor of the MsbA multicopy refinement was not outrageously high com­pared with other PDB files of comparable resolution: as of December 2007, crystal structures in the PDB with resolutions between 4.0 and 4.5 Å had a mean free *R* factor of 33% with a wide range of variation between 22 and 45%. The free *R* factor of the single-model refinement lay at the upper end of this range (Table 2 ▶), which should have been a warning sign.

Of particular interest is the observation that even with the corrected structures the free *R*-factor values for the single model are sometimes similar to those of multicopy refinements of the incorrect structures (Tables 3 ▶ and 4 ▶). The combination of noncrystallographic symmetry and multicopy refinement substantially compromised the effectiveness of the free *R* factor as a measure of structure quality. This suggests that in at least some cases the free *R* factor has its limitations as a structure-quality indicator and is vulnerable to a certain amount of manipulation to achieve values that would allow publication. The agreement of the model with experimental and model-phased electron density is an extremely important factor that was neglected in this case and which is less trivial to present in a paper.

Although each subsequent structure of MsbA and EmrE was associated with experimental phases (Chang, 2003 ▶; Reyes & Chang, 2005 ▶; Pornillos *et al.*, 2005 ▶), it is evident that the previously determined topologies were biasing the interpretation of the subsequent experimental electron-density maps. It is noteworthy that the subsequent structures were not determined by molecular replacement, despite the availability of these prior structures.

### 5.1. Conserved substructures

One possibility to test for incorrect structures utilizes domains or subdomains of conserved structure. Such potential existed in MsbA, where the ATPase domain was similar to that of the ATPase domain of *S. typhimurium* histidine permease (HisP; Hung *et al.*, 1998 ▶). For *E. coli* MsbA, alignments of HisP onto the structurally homologous domain gave a root-mean-square deviation (r.m.s.d.) of 1.5 Å for 90 equivalent C^α^ atoms for the correct structure. For the incorrect structure the same alignment procedure gave an r.m.s.d. of 1.6 Å for 44 C^α^ atoms. However, the converse is true for the case of *S. typhimurium* MsbA: the correct structure has an r.m.s.d. of 1.5 Å for 176 equivalent C^α^ atoms and the incorrect structure has an r.m.s.d. of 1.3 Å for 195 C^α^ atoms. This test does not prove to be sensitive for an incorrect structure, although the result may be biased because the sequence homology was known before the models of MsbA were built.

### 5.2. Validation and data deposition

Critical assessment of structure quality requires access to all the coordinates and at a minimum to the native data used during refinement (Jones & Kleywegt, 2007 ▶; Joosten & Vriend, 2007 ▶). This policy has been actively advocated for a number of years, yet some of the MsbA and EmrE structures were deposited as only the C^α^ atoms, including the coordinate sets corresponding to the corrected structures (Chen *et al.*, 2007 ▶; Ward *et al.*, 2007 ▶). This substantially undermines the ability to independently assess structure quality, as was clearly necessary in this case.

## 6. Conclusions

The original error causing the inversion of the anomalous sign was a simple mistake (Chang *et al.*, 2006 ▶). The MAD phasing examples in this paper demonstrate that it is trivial to propagate this error to the point of generating an experimental electron-density map with superficially acceptable phasing statistics. A straightforward way to minimize these types of errors is to make use of widely deployed and well tested program suites, as exemplified by the *CCP*4 suite itself (Collaborative Computational Project, Number 4, 1994 ▶).

The error was independently repeated for both the MsbA and EmrE structures and should have been caught at the point of map interpretation. The low resolution of the structural studies hampered the interpretation, but these resolutions are by no means unprecedented. It is not easy to understand how the error evaded detection over the course of five papers in four years (Chang & Roth, 2001 ▶; Chang, 2003 ▶; Ma & Chang, 2004 ▶; Reyes & Chang, 2005 ▶; Pornillos *et al.*, 2005 ▶) even if one allows that existing structures bias the interpretation of subsequent structures. The aggressive use of novel refinement techniques, an ill-advised method of selecting test-set reflections and the habit of truncating the data in refinement at 2σ(*F*) to remove the weak data all played a role in giving rise to acceptable refinement statistics (Table 2 ▶). A focus on getting the free *R* factor into the ‘publishable range’ appears to have been a larger factor than careful assessment of how the model fitted the experimental electron density.

Low-resolution structures are especially challenging from a technical point of view, but are often very rewarding in the amount of biological insight that they reveal. In the case of MsbA and EmrE the potential impact of the structures appears to have overwhelmed considerations as to their accuracy. Despite advancing standards and tools for quality control of macromolecular structures, the fundamentally incorrect atomic models of MsbA and EmrE made their way into the literature multiple times. The impact of these incorrect structures has extended beyond embarrassment for the scientists concerned and affected research in other laboratories (Petsko, 2007 ▶).

Ultimately, the incorrect structures of MsbA and EmrE were uncovered by the careful work of others (Dawson & Locher, 2006 ▶; Tate, 2006 ▶). The lesson for the rest of us may well be that we can never take too much care with our own structure determinations, lest we attract equally unflattering attention.

## Figures and Tables

**Table 1:** Results of MAD phasing test cases Values in bold indicate solutions that give rise to interpretable electron-density maps. Statistics reported from substructure solution using *SHELXD* (Schneider & Sheldrick, 2002 ▶) and *SHELXE* (Sheldrick, 2008 ▶).

	MC159		S2P	
	Δ_ano_	−Δ_ano_	Δ_ano_	−Δ_ano_
*SHELXD*				
Patterson figure of merit	13.70	13.13	21.21	22.26
Correlation on all/weak (%)	42.03/30.45	40.64/28.74	38.06/27.15	36.06/24.49
*SHELXE*				
Contrast	0.32	0.32	0.57	**0.76**
Pseudo-free correlation (%)	53.0	57.8	60.4	**64.3**
*SHELXE* (sites inverted)				
Contrast	**0.49**	**0.49**	**0.76**	0.55
Pseudo-free correlation (%)	**73.5**	**73.6**	**64.8**	59.0

**Table 2:** MsbA and EmrE refinement statistics for the incorrect structures Results of crystallographic refinement as reported for MsbA and EmrE structures (Chang & Roth, 2001 ▶; Chang, 2003 ▶; Reyes & Chang, 2005 ▶; Ma & Chang, 2004 ▶; Pornillos *et al.*, 2005 ▶).

	*E. coli* MsbA		*S. typhimurium* MsbA	*Vibrio cholera* MsbA		EmrE–TPP	Apo EmrE
Models	Single	Multicopy	Multicopy	Single	Multicopy	Multicopy	Multicopy
Resolution (Å)	4.5		4.2	3.8		3.7	3.8
*R* factor† (%)	38	27	28	38	24	28	32
Free *R* factor† (%)	45	38	33	41	33	35	35
Test-set selection	Random		Random	Random		Random	Random
Data cutoff	0σ(*F*)		nd	2σ(*F*)		2σ(*F*)	2σ(*F*)
R.m.s.d. bonds‡ (Å)	0.009		0.006	0.01		0.03	0.01
Average *B* value§ (Å^2^)	90		80	90		40	55
Asymmetric unit	8 monomers		2 dimers	4 monomers		1 dimer	2 tetramers
PDB code	1jsq		1z2r	1pf4		2f2m	1s7b

† The *R* factor is *R* = 
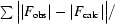
 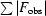 for working-set data; the free *R* factor is the same quantity calculated for the test-set reflections.

‡ Root-mean-square deviation between ideal and observed bond-length stereochemistry.

§ Average *B* value of the model.

**Table 3:** Comparison of incorrect and correct MsbA models Results of crystallographic refinement as reported for MsbA structures (Chang & Roth, 2001 ▶; Chang, 2003 ▶; Reyes & Chang, 2005 ▶; Ward *et al.*, 2007 ▶). Completeness and *R*-factor values in parentheses were calculated with a 2σ(*F*) cutoff.

	*E. coli* MsbA			*S. typhimurium* MsbA	
Space group	*P*1			*C*2	
Model†	Wrong-S	Wrong-M	Right	Wrong-M	Right
Resolution (Å)	4.5		5.3	4.2	4.2
Completeness (%)	nd		97 (65)	nd	86 (78)
*R* factor‡ (%)	38	27	28 (24)	28	34 (32)
Free *R* factor‡ (%)	45	38	31 (28)	33	36 (35)
Free *R* − *R* (%)	7	11	3	5	2
Test-set selection	Random		Random	Random	Random
Data cutoff	0σ(*F*)		0σ(*F*) [2σ(*F*)]	nd	0σ(*F*) [2σ(*F*)]
R.m.s.d. bonds§ (Å)	0.009		0.008	0.006	0.012
Average *B* factor¶ (Å^2^)	90		278	80	156
Ramachandran plot†† (%)	—		—	73	—
PDB code	1jsq		3b5w	1z2r	3b5z

† Wrong-M refers to multicopy refinement of the incorrect structure and Wrong-S refers to refinement of a single-copy model of the incorrect structure.

‡ The *R* factor is *R* = 
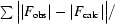
 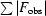 for working-set data; the free *R* factor is the same quantity calculated for the test-set reflections.

§ Root-mean-square deviation between ideal and observed bond-length stereochemistry.

¶ Average *B* value of the model.

†† Proportion of residues lying in the most favored region of the Ramachandran plot as calculated by *PROCHECK* (Laskowski *et al.*, 1993 ▶).

**Table 4:** Comparison of incorrect and correct EmrE models Results of crystallographic refinement as reported for EmrE structures (Ma & Chang, 2004 ▶; Pornillos *et al.*, 2005 ▶; Chen *et al.*, 2007 ▶).

	EmrE–TPP		Apo EmrE	
Space group	*C*2		*F*222	
Model†	Wrong-M	Right	Wrong-M	Right
Resolution (Å)	3.7	4.0	3.8	4.2
Completeness (%)	98	41	nd	86
*R* factor[Table-fn tfn10] (%)	28	33	32	34
Free *R* factor[Table-fn tfn10] (%)	35	36	35	36
Free *R* − *R*	7	3	3	2
Test-set selection	Random	nd	Random	Random
Data cutoff	2σ(*F*)	0σ(*F*)	2σ(*F*)	0σ(*F*)
R.m.s.d. bonds[Table-fn tfn11] (Å)	0.030	0.013	0.010	0.013
Average *B* factor[Table-fn tfn12] (Å^2^)	40	393	55	150
Ramachandran plot[Table-fn tfn13] (%)	82	—	76	—
PDB code	2f2m	—	1s7b	3b61

† Wrong-M refers to multicopy refinement of the incorrect structure.

‡ The *R* factor is *R* = 
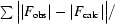
 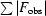 for working-set data; the free *R* factor is the same quantity calculated for the test-set reflections.

§ Root-mean-square deviation between ideal and observed bond-length stereochemistry.

¶ Average *B* value of the model.

†† Proportion of residues lying in the most favored region of the Ramachandran plot as calculated by *PROCHECK* (Laskowski *et al.*, 1993 ▶).

## References

[bb1] Berman, H. M., Westbrook, J., Feng, Z., Gilliland, G., Bhat, T. N., Weissig, H., Shindyalov, I. N. & Bourne, P. E. (2000). *Nucleic Acids Res.* **28**, 235–242. 10.1093/nar/28.1.235 PMC102472 10592235

[bb2] Brünger, A. T. (1997). *Methods Enzymol.* **277**, 366–396. 10.1016/s0076-6879(97)77021-6 18488318

[bb3] Chang, G. (2003). *J. Mol. Biol.* **330**, 419–430. 10.1016/j.jmb.2003.05.001 17580380

[bb4] Chang, G. (2007). *J. Mol. Biol.* **369**, 596. 10.1016/j.jmb.2003.05.001 17580380

[bb5] Chang, G. & Roth, C. B. (2001). *Science*, **293**, 1793–1800. 10.1126/science.293.5536.1793 11546864

[bb6] Chang, G., Roth, C. B., Reyes, C. L., Pornillos, O., Chen, Y. J. & Chen, A. P. (2006). *Science*, **314**, 1875. 10.1126/science.314.5807.1875b 17185584

[bb8] Chen, Y. J., Pornillos, O., Lieu, S., Ma, C., Chen, A. P. & Chang, G. (2007). *Proc. Natl Acad. Sci. USA*, **104**, 18999–19004. 10.1073/pnas.0709387104 PMC2141897 18024586

[bb7] Chen, Z. & Chapman, M. S. (2001). *Biophys. J.* **80**, 1466–1472. 10.1016/S0006-3495(01)76118-8 PMC1301337 11222306

[bb9] Collaborative Computational Project, Number 4 (1994). *Acta Cryst.* D**50**, 760–763.

[bb19] Dawson, R. J. & Locher, K. P. (2006). *Nature (London)*, **443**, 180–185. 10.1038/nature05155 16943773

[bb10] Fabiola, F., Korostelev, A. & Chapman, M. S. (2006). *Acta Cryst.* D**62**, 227–238. 10.1107/S0907444905040680 16510969

[bb11] Feng, L., Yan, H., Wu, Z., Yan, N., Wang, Z., Jeffrey, P. D. & Shi, Y. (2007). *Science*, **318**, 1608–1612. 10.1126/science.1150755 18063795

[bb12] Hung, L.-W., Wang, I. X., Nikaido, K., Liu, P.-Q., Ames, G. F.-L. & Kim, S.-H. (1998). *Nature (London)*, **396**, 703–707. 10.1038/25393 9872322

[bb13] Hunte, C., Screpanti, E., Venturi, M., Rimon, A., Padan, E. & Michel, H. (2005). *Nature (London)*, **435**, 1197–1202. 10.1038/nature03692 15988517

[bb14] Jones, T. A. & Kleywegt, G. J. (2007). *Science*, **317**, 194–195. 10.1126/science.317.5835.194c 17626864

[bb15] Joosten, R. P. & Vriend, G. (2007). *Science*, **317**, 195–196. 10.1126/science.317.5835.195 17626865

[bb16] Kraulis, P. J. (1991). *J. Appl. Cryst.* **24**, 946–950.

[bb17] La Fortelle, E. de & Bricogne, G. (1997). *Methods Enzymol.* **276**, 472–494. 10.1016/S0076-6879(97)76073-7 27799110

[bb20] Laskowski, R. A., MacArthur, M. W., Moss, D. S. & Thornton, J. M. (1993). *J. Appl. Cryst.* **26**, 283–291.

[bb18] Li, F. Y., Jeffrey, P. D., Wu, J. W. & Shi, Y. (2006). *J. Biol. Chem* **281**, 2960–2968. 10.1074/jbc.M511074200 16317000

[bb21] Ma, C. & Chang, G. (2004). *Proc. Natl. Acad. Sci. USA*, **101**, 2852–2857.

[bb22] Ma, C. & Chang, G. (2007). *Proc. Natl. Acad. Sci. USA*, **104**, 3668.

[bb23] Matthews, B. W. (2007). *Protein Sci.* **16**, 1013–1016. 10.1110/ps.072888607 PMC2206657 17473006

[bb24] Merritt, E. A. & Murphy, M. E. P. (1994). *Acta Cryst.* D**50**, 869–873. 10.1107/S0907444994006396 15299354

[bb25] Miller, C. (2007). *Science*, **315**, 459. 10.1126/science.315.5811.459b 17255494

[bb26] Miller, G. (2006). *Science*, **314**, 1856–1857. 10.1126/science.314.5807.1856 17185570

[bb27] Otwinowski, Z. & Minor, W. (1997). *Methods Enzymol.* **276**, 307–326. 10.1016/S0076-6879(97)76066-X 27754618

[bb28] Pellegrini, M., Gronbech-Jensen, N., Kelly, J. A., Pfleugel, G. M. & Yeates, T. O. (1997). *Proteins*, **29**, 426–432. 10.1002/(sici)1097-0134(199712)29:4<426::aid-prot3>3.0.co;2-6 9408940

[bb29] Perrakis, A., Morris, R. M. & Lamzin, V. S. (1999). *Nature Struct. Biol.* **6**, 458–463. 10.1038/8263 10331874

[bb30] Petsko, G. A. (2007). *Genome Biol.* **8**, 103. 10.1186/gb-2007-8-2-103 PMC1852413 17328789

[bb31] Pornillos, O., Chen, Y. J., Chen, A. P. & Chang, G. (2005). *Science*, **310**, 1950–1953. 10.1126/science.1119776 16373573

[bb32] Reyes, C. L. & Chang, G. (2005). *Science*, **308**, 1028–1031. 10.1126/science.1107733 15890884

[bb33] Schneider, T. R. & Sheldrick, G. M. (2002). *Acta Cryst.* D**58**, 1772–1779. 10.1107/s0907444902011678 12351820

[bb35] Sheldrick, G. M. (2008). *Acta Cryst.* A**64**, 112–122. 10.1107/S0108767307043930 18156677

[bb36] Storoni, L. C., McCoy, A. J. & Read, R. J. (2004). *Acta Cryst.* D**60**, 432–438. 10.1107/S0907444903028956 14993666

[bb37] Tate, C. G. (2006). *Curr. Opin. Struct. Biol.* **16**, 457–464. 10.1016/j.sbi.2006.06.005 16828280

[bb38] Terwilliger, T. C. & Berendzen, J. (1999). *Acta Cryst.* D**55**, 849–861. 10.1107/S0907444999000839 PMC2746121 10089316

[bb34] Wang, J., Wlodawer, A. & Dauter, Z. (2007). *Acta Cryst.* D**63**, 751–758. 10.1107/S0907444907025620 17582166

[bb39] Ward, A., Reyes, C. L., Yu, J., Roth, C. B. & Chang, G. (2007). *Proc. Natl Acad. Sci. USA*, **104**, 19005–19010. 10.1073/pnas.0709388104 PMC2141898 18024585

